# A simulation framework for evaluating multi-stage sampling designs in populations with spatially structured traits

**DOI:** 10.7717/peerj.6471

**Published:** 2019-02-25

**Authors:** Patricia Puerta, Lorenzo Ciannelli, Bethany Johnson

**Affiliations:** 1 College of Earth, Ocean, and Atmospheric Sciences, Oregon State University, Corvallis, OR, USA; 2 Instituto Español de Oceanografía, Centro Oceanográfico de Baleares, Palma de Mallorca, Spain; 3 Applied Mathematics, Baskin School of Engineering, University of California Santa Cruz, Santa Cruz, CA, USA

**Keywords:** Sampling design, Multi-stage sampling, Life history traits, Spatial ecology, Population ecology

## Abstract

Selecting an appropriate and efficient sampling strategy in biological surveys is a major concern in ecological research, particularly when the population abundance and individual traits of the sampled population are highly structured over space. Multi-stage sampling designs typically present sampling sites as primary units. However, to collect trait data, such as age or maturity, only a sub-sample of individuals collected in the sampling site is retained. Therefore, not only the sampling design, but also the sub-sampling strategy can have a major impact on important population estimates, commonly used as reference points for management and conservation. We developed a simulation framework to evaluate sub-sampling strategies from multi-stage biological surveys. Specifically, we compare quantitatively precision and bias of the population estimates obtained using two common but contrasting sub-sampling strategies: the random and the stratified designs. The sub-sampling strategy evaluation was applied to age data collection of a virtual fish population that has the same statistical and biological characteristics of the Eastern Bering Sea population of Pacific cod. The simulation scheme allowed us to incorporate contributions of several sources of error and to analyze the sensitivity of the different strategies in the population estimates. We found that, on average across all scenarios tested, the main differences between sub-sampling designs arise from the inability of the stratified design to reproduce spatial patterns of the individual traits. However, differences between the sub-sampling strategies in other population estimates may be small, particularly when large sub-sample sizes are used. On isolated scenarios (representative of specific environmental or demographic conditions), the random sub-sampling provided better precision in all population estimates analyzed. The sensitivity analysis revealed the important contribution of spatial autocorrelation in the error of population trait estimates, regardless of the sub-sampling design. This framework will be a useful tool for monitoring and assessment of natural populations with spatially structured traits in multi-stage sampling designs.

## Introduction

Comparisons of different sampling strategies used to collect biological data have been performed in multiple fields such as forestry (Kulow, 1966; Gove, Ducey & Valentine, 2002; Broich et al., 2009; Bhatta, Chaudhary & Vetaas, 2012); grasslands and crops (Colbach, Dessaint & Forcella, 2000; Stafford et al., 2006); land-use (Nusser et al., 2013); terrestrial mammals (Parmenter et al., 2003; Harris et al., 2013; Wright, Newson & Noble, 2014; Calmanti et al., 2015); birds (Johnson et al., 2009; Pavlacky et al., 2017); marine invertebrates (Miller & Ambrose, 2000; Cole et al., 2001; Li et al., 2015); and fish (Kimura, 1977; Lai, 1993; Goodyear, 1995; Liu, Chen & Cheng, 2009). These comparisons identify an optimal design that balances sampling effort and data quality to produce accurate estimates of the studied population parameters. Usually, the main objective of the sampling design is obtaining unbiased and precise estimates of some population parameters such as total abundance, which is one of the critical reference values for management and conservation. However, many marine, terrestrial and freshwater populations vary across space and time not only on abundance values, but also on traits of the composing individuals, such as length, maturity, survival. These traits affect important assessment products, such as the age composition and structure of a population and ultimately estimates of population abundance (Le Page & Cury, 1995; Childs et al., 2003; Metcalf et al., 2009; Betti, Wahl & Zamir, 2016).

In many cases, the necessary technical procedures to determine trait values from individuals collected are costly and time consuming and thus, not all individuals in the sampled population can be analyzed (Kimura, 1977; Castro & Lawing, 1995; Matsumura, Arlinghaus & Dieckmann, 2012; Hof & Bright, 2016). As a consequence, ecologists frequently need to apply a multi-stage design to collect data of a particular trait and thus, monitor a biological population. Sampling sites are the primary units in the multi-stage design, while the trait data are collected as a sub-sample in each site. For instance, multi-stage design is very common in the monitoring of fish populations, whereby fish are caught at different sampling sites, all or a high proportion of the fish in that catch are measured for length, but only a sub-sample of the measured fish are aged. Comparisons of different sub-sampling designs for collecting and studying the distribution of a particular trait, such as age composition of a populations (Kimura, 1977; Pennington, Burmeister & Hjellvik, 2002; Chih, 2009; Hulson, Hanselman & Shotwell, 2016), have been more thoroughly studied in fisheries science than in other ecology fields (Kosmelj, Cedilnik & Kalan, 2001; Stafford et al., 2006; Smith et al., 2011; Brown et al., 2013). Thus, the sampling design to gather data on population abundance (stage 1) is paramount in ecological research, but also the sub-sampling design to gather data on traits-structure in the population (stage 2 or 3) can have a major impact on the population estimates, which will be used as reference points for assessment and management. The potential bias and precision of the estimates derived from different sub-sampling designs in multi-stage samplings have rarely been evaluated to date (Bassett & Edwards, 2003; Rhodes & Jonzén, 2011). This is particularly problematic when the individual traits within a population are structured across space and time, calling into question the statistical representativeness of the subsample.

Computer simulations are an essential approach in ecological research for comparing and evaluating different sampling methods (Legendre et al., 2002; Harrison et al., 2013; Cao et al., 2014; Parker et al., 2016). First, in contrast to empirical studies of natural populations, simulation studies have the advantage that the true parameter values are known. Second, virtual populations can mimic a variety of scenarios, each differing in terms of biological (abundance, age structure, etc.) and statistical (spatial distribution, autocorrelation, trends, etc.) attributes, making any conclusion reasonably applicable to a variety of natural conditions. Third, alternative sub-sampling designs can be compared quantitatively with respect to the accuracy, precision and bias of the population estimates obtained (Legendre et al., 2002; Anderson et al., 2014; Parker et al., 2016).

Here, we developed a simulation framework for a sub-sampling strategy evaluation in specific trait data collections obtained from multi-stage biological surveys. For this purpose, we compared two commonly used but contrasting sub-sampling strategies in the collection of trait data in fish populations: the random and the stratified designs. Using the stratified strategy, the sampler collects a specific number of individuals at every pre-defined stratum, interval or group (for example depth strata or length interval) to assess the population trait patterns. By contrast, using the random strategy (RS), the sampler simply obtains a particular number of individuals randomly regardless of any other conditions. We applied our simulation framework to the Pacific cod (*Gadus macrocephalus*) population of the Eastern Bering Sea (EBS) (Thompson, 2017). The simulation presented here is reproducible and broadly applicable to compare and evaluate sub-sampling strategies in any biological data collection under a multi-stage design and is particularly applicable when the population presents spatially or temporally structured traits. This framework might help to select appropriate and efficient (sub-) sampling designs to improve data quality in ecological research and assessment.

## Materials and Methods

To develop a simulation framework for a sub-sampling strategy evaluation in spatially structured populations the following steps are required: (1) create a virtual population where all the parameters and features of interest are known; (2) simulate a multi-stage field sampling for each (sub-) sampling design targeted; (3) account for further sample and data processing for the trait estimates by addressing and adding potential bias and errors resulting from those proceedings; (4) compare and evaluate the estimates obtained from each (sub-) sampling strategy and finally; (5) perform a sensitivity test to quantify errors in the population estimates and disentangle error sources from the (sub-) sampling design or other processes such as those included in step 3. We provide below a detailed description of each of the five steps within the biological context of our case of study: evaluation of random and length-stratified sub-sampling strategies for the collection of age data in the EBS Pacific cod population.

## Case Study: Eastern Bering Sea Pacific Cod

Pacific cod (*Gadus macrocephalus*) is distributed across the entire continental shelf of the EBS and is thought to be a single population. The EBS continental shelf extends over more than 500 km with a steep shelf break in the western boundary. The shelf is divided into inner (<50 m depth), middle (50–100 m) and outer (100–200 m) regions considering bathymetry and oceanographic characteristics (Coachman, 1986; Stabeno et al., 2001). Pacific cod typically move from inner to outer shelf as they age, displaying a progressive increase in mean length across space. Additionally, spatial variation in size at age was observed with a difference up to five cm between fish of the same age inhabiting inner and outer shelf (Puerta et al., 2018). Females tend to be a few cm larger than males, particularly in old individuals. EBS Pacific cod reproduces once a year, which typically gives rise to a multimodal length distribution, where each mode corresponds to a young age-group. The inter-annual variability in spatial distribution of the Pacific cod across the EBS shelf is particularly influenced by the extension of the “cold pool.” This is a water layer <2 °C formed from winter sea ice, which induces cooling and increase salinity and density of the surface water and the subsequent spring melt, thus inducing thermal stratification of the middle shelf (Stabeno et al., 2001). It may act as a cross-shelf migration barrier for subarctic fish species, forcing part of the population to remain on the outer shelf (Ciannelli & Bailey, 2005; Kotwicki et al., 2005).

The assessment and management of the EBS Pacific cod stock relies to a considerable extent on data from scientific bottom trawl surveys pertaining to the relative abundance and age and length structure of the population (Thompson, 2017). Relative abundance and length composition data have been collected annually since 1982, and survey age composition data are available from 1994 to 2016 (Conner & Lauth, 2017), following a multi-stage sampling design to record abundance, length and age data. The survey follows a square grid pattern sampling 375 sites and performs a similar cruise route every year that starts in the SE inner-middle shelf, then moves toward the NW region. At any given sampling site (stage 1), all or a high percentage of the fish caught is measured for length (stage 2). However, only a fraction of the measured fish is collected for ageing due to the process being costly and time-consuming (stage 3). Through 2016, this final sub-sample for age data followed a length-stratified design; that is three fish at each given length interval (one cm), sex and area (NW and SE) were collected. This sub-sampling strategy ensures a wide coverage of age composition, but might misrepresent spatial patterns in size and age since once the number of fish for a given length interval is covered, no more fish at that length interval are collected in the survey (Puerta et al., 2018).

### Virtual population

We simulated a virtual population by resampling the observed data from surveys to preserve the spatio-temporal and statistical features of the natural population. Since natural populations are dynamic and change over time and space in response to the variations in their environment, the virtual population encompasses biological data under different demographic and physical conditions. Thus, we generated six scenarios, which when compiled represent the average physical and demographic conditions of the virtual population. The six scenarios conforming the average virtual population included (1) warm and (2) cold scenarios, based on the average bottom temperature of the middle shelf, since the spatial distribution is highly influenced by water temperature (Stabeno et al., 2001; Ciannelli & Bailey, 2005; Barbeaux, 2017). To assess the studied trait structure, we generated two more scenarios corresponding to (3) high and (4) low age structure diversity. Diversity with respect to age structure was measured using the *Simpsons diversity index* in the *vegan* (Oksanen et al., 2018) library of R software (R Core Team, 2017). The population abundance (stage 1) and number of fish measured (stage 2) in the high and low age diversity scenarios were set to the same values to avoid confounding this effect with those of abundance variability. Finally, we incorporated (5) high and (6) low total abundance scenarios into the virtual population. In order to prevent similar confounding effects as aforementioned, the age structures of these two scenarios were constrained to be as similar as possible by keeping less than a 3% difference in the number of individuals at any given age-group.

To create every particular scenario (for instance, high total abundance), the resampling was limited to the observed data that meet the condition of that particular scenario (for instance, data on the 5 years with the highest total abundance). For each scenario, we calculated total abundance (stage 1) and percentage of individuals sub-sampled for trait analysis (stages 2–3) at any given sampling location. Further, we determined a value of the trait of interest for every individual in the virtual population using a function to estimate this trait. The parameters of the trait function can be tuned to the observed data to represent the realized spatial variability of individual traits. Populations spatially structured in abundance or traits display some degree of spatial autocorrelation across locations, which was also included in the virtual population.

The virtual population included, by site, as many fish as those historically measured on average over the years corresponding to a particular scenario (except for the high and low age diversity scenarios, which share the same values as aforementioned). Since the sex of some fish was undetermined in the observed data, we assigned sex to each fish based on the sex ratio calculated for each length interval (one cm) and site. When the sample size was lower than 10 fish, the sex ratio was calculated by a higher level of aggregation such as length interval and stratum, only length interval and finally, overall sex ratio. Further, we determined the age of every fish based on its length and the geographic location where it was caught (i.e., sampling site), using the following Eq. (1):

(1) $${\rm{Ag}}{{\rm{e}}_{i\;}} = \;{\rm{\alpha }} + \;{{\rm{\beta }}_1}\;{\rm{Size}}_i^2 + \;{{\rm{\beta }}_{2{\rm{\;}}}}r{{\rm{\lambda }}_i} + {e_i}$$

where *i* corresponds to every sampled fish, α is the intercept, β_1_ is the size coefficient, which varies between male (1.05 *e*^−5^) and females (1 *e*^−5^) and β_2_ is the geographic coefficient (−0.031) that accounts for size at age variability in rotated latitude (*r*λ) across the shelf. Due to the NW—SE orientation of the EBS inner-outer shelf axis, geographical coordinates were rotated to accommodate and simplify this orientation to a N—S axis. We added a site-specific spatial auto-correlated error (*e_i_*) to the age values obtained in Eq. (1). Spatial auto-correlation among sampling sites was calculated from a covariogram based on sampling site coordinates and average age residuals from observed data using *gstat* (Pebesma, 2004) and *fields* (Nychka et al., 2017) libraries in R software (R Core Team, 2017).

### Sampling simulation

We simulated a multi-stage field sampling in every scenario of the virtual population and for each of the (sub-) sampling designs under evaluation. This was equivalent to conducting a field survey in the study area for as many times as the number of scenarios. Sampling of the virtual population (stage 1) occurred in all of the sampling sites for each of the six scenarios, resembling the same protocol used in the scientific surveys (Conner & Lauth, 2017; Puerta et al., 2018). At half of the sampling sites (randomly selected), two different sub-sampling strategies for age data collection (stage 3) were simulated to be compared: the length-stratified strategy (LSS), that collects three fish per length interval (one cm), sex and area (NW or SE of the EBS); and the RS, which randomly collects four fish at each site, regardless of the length, sex or area. Thus, the total sample size of the RS is larger than LSS. These sample sizes represent the minimum effort and were chosen to closely mimic the current sampling protocols in the Pacific cod case study. We also assessed results in which the stage 3 sample size was equal to all fish caught (i.e., no subsampling) and results where LSS and RS ended up in the same sample size. For the latter, and for each scenario, several records in the RS were randomly removed to match the total sample size obtained with the LSS.

### Simulation of trait sub-sample processing

Characterization of some important individual traits such as age or maturity relies on statistical estimations from technical proceedings and/or depends on subjective interpretations of the biological conditions since the true value of the trait is usually unknown. Thus, the process of extracting trait information from the collected biological sub-samples introduces a new component of variability that includes bias and error from the trait’s true value.

Ageing processes in fish mainly relies on the interpretation of annual marks (annuli) in the otoliths. Despite otolith readers being expertly trained, some degree of subjectivity is involved (Matta & Kimura, 2012). Kastelle et al. (2017) defined age-specific bias in EBS Pacific cod ageing by comparing ages derived from counting otolith marks and those (considered true ages) derived from stable oxygen isotopes (δ^18^O) analysis. Overall, the probability of assigning an age equal to the true age was approximately 61% (Kastelle et al., 2017). In contrast, the error in otolith reading refers to the degree to which an age estimate is reproducible by the same or a different reader (Matta & Kimura, 2012). Usually a second reader tests 20% of all specimens to calculate the age-specific inter-reader coefficient of variation (Kimura & Anderl, 2005; Matta & Kimura, 2012). Thus, in our case study simulation, we differentiate the “true age” of an individual (calculated from Eq. (1) in the virtual population) from the “otolith age,” which is recorded in the age sub-sample and derived from the true age plus the age-specific reading bias and error (Kastelle et al., 2017).

### Evaluation of the (sub-) sampling strategy

We followed a (sub-) sampling strategy evaluation based on the comparison of: (i) prediction errors for the trait values derived from a statistical model, with the same covariate as those included in Eq. (1), parameterized on each of the trait sub-samples, respectively; (ii) the trait frequency distribution of the virtual population estimated from each sub-sample; (iii) the mean and modal estimated values of the trait and; (iv) the average spatial patterns of the trait recovered from each sub-sample. This approach ensures a quantitatively valid comparison of the estimates derived from each (sub-) sampling strategy.

- We used generalized additive models (GAM; Wood, 2006) to fit “otolith age” (as described above) in relation to fish length, sex and geographical location (Puerta et al., 2018), using *mgcv* library (Wood, 2006) in R software (R Core Team, 2017). Model Eq. (2) was defined as follows,
  (2) $${\rm{Ag}}{{\rm{e}}_i} = {\rm{\alpha }}\; + \;{s_1}\left({{\rm{Siz}}{{\rm{e}}_i}} \right) \times \;{\rm{Se}}{{\rm{x}}_i} + {s_2}\left({{\rm\phi _i},{{\rm{\lambda }}_i}} \right) + \;{{\rm{\varepsilon }}_i}$$
  where *i* corresponds to every sampled fish. α is an intercept, and *s* represents the smooth functions for the *Length* and the geographic location (longitude φ, latitude λ). To reduce circularity, the model Eq. (2) is purposely different from Eq. (1) (used to predict the true age of individuals in the virtual population). *Sex* is included as a factor, and so, different smooth functions are fitted for males and females; and ε is a normally distributed error term. Since the RS and LSS age sub-samples are by definition not proportional to the catch, these data were weighted by the size-specific abundance value within each site in the model. This ensures that age data from a particular length interval with a higher representation in the catch (as number of fish) have larger weights in the model. Additionally, these weights correct variance structure in multistage data, removing any bias in the precision of model estimates (Zuur et al., 2009). The mean predicted errors of the models (as model “otolith age” prediction minus virtual population “true age”) parameterized on the LSS and RS sub-samples were compared as an indicator of goodness of fit and predictability skills. The mean predicted error was calculated on 300 iterations by removing 30% of the data at a time and predicting age of the deleted cases with a model fitted on the remaining data.
- Then, we used “otolith age” predictions of the Eq. (2) model parameterized on each age (sub-) sampling strategy sample to estimate the age frequency distribution of the virtual population.
- Mean and modal size at age obtained from the LSS and RS age sub-samples, respectively, were compared for age-groups 1 to 3. Finally, (iv) spatial patterns in average size and age over the EBS shelf derived from the LSS and RS age sub-samples were compared with those of the virtual population.

### Sensitivity test

We performed a sensitivity test to disentangle and quantify (i) the contribution of the spatial term included in the model used in the sub-sampling strategy evaluation (Eq. (2)), (ii) source and magnitude of the errors in the population estimates derived from the inclusion of spatial autocorrelation in the virtual population and (iii) bias and error derived from trait sub-sample processing. The aforementioned evaluation was repeated for a Pacific cod virtual population without spatial autocorrelation and RS and LSS age sub-samples where the “otolith age” equals the “true age,” and every other combination of these errors to compare and quantify error contribution in the population estimates.

## Results

### Virtual population

**Figure 1:**
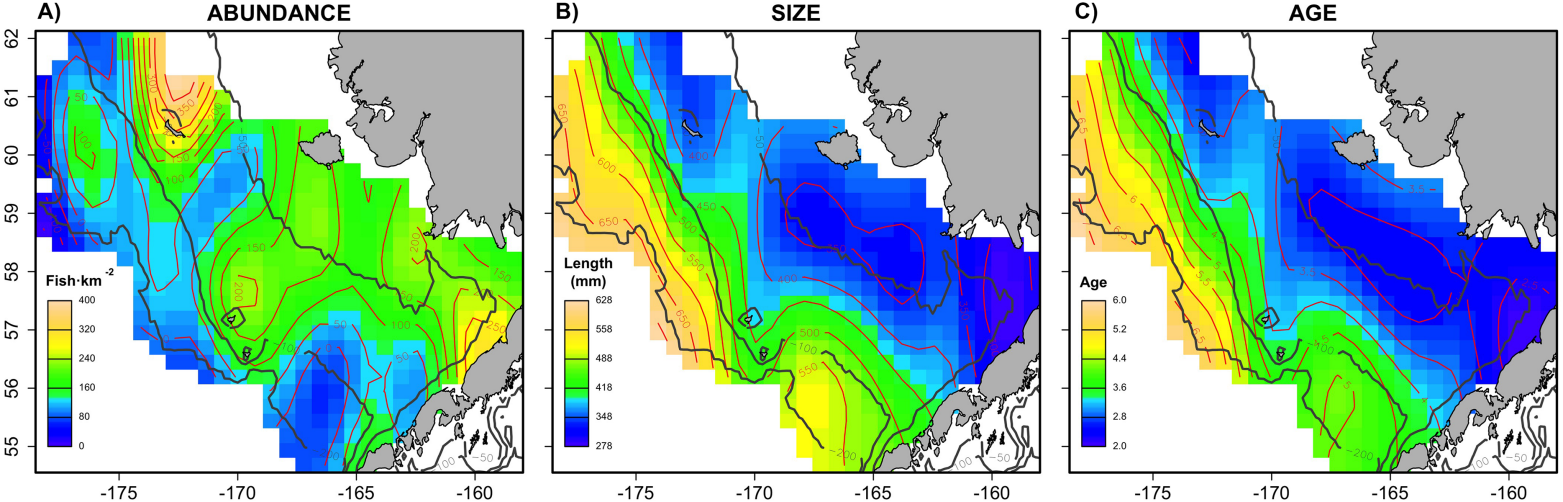
Spatial patterns of the virtual population. Spatial distribution of (A) abundance, (B) size and (C) age of the virtual population, showing average patterns combining the six scenarios.

The combined Pacific cod virtual population included approximately 83,000 individuals across the six simulated scenarios, with an average of 13,833 individuals simulated per sampling event (i.e., survey year). The spatial distribution (Fig. 1) in abundance averaged across the six scenarios highlights northern and southern regions in the inner shelf as the hotspot in the study area, although the central middle shelf also presented remarkable abundance. Size and age exhibited similar average spatial patterns, increasing progressively from the inner to the outer continental shelf. Larger and older individuals were particularly concentrated in the northwest region of the outer shelf. However, the distributional patterns in abundance, size and age varied considerably across the six individual scenarios (Fig. S1). For example, in warm years cod abundance is higher in the inner and middle shelf of the Bering Sea, and average age and size is less structured over space than in cold years.

### Evaluation of the (sub-) sampling strategies

**Figure 2:**
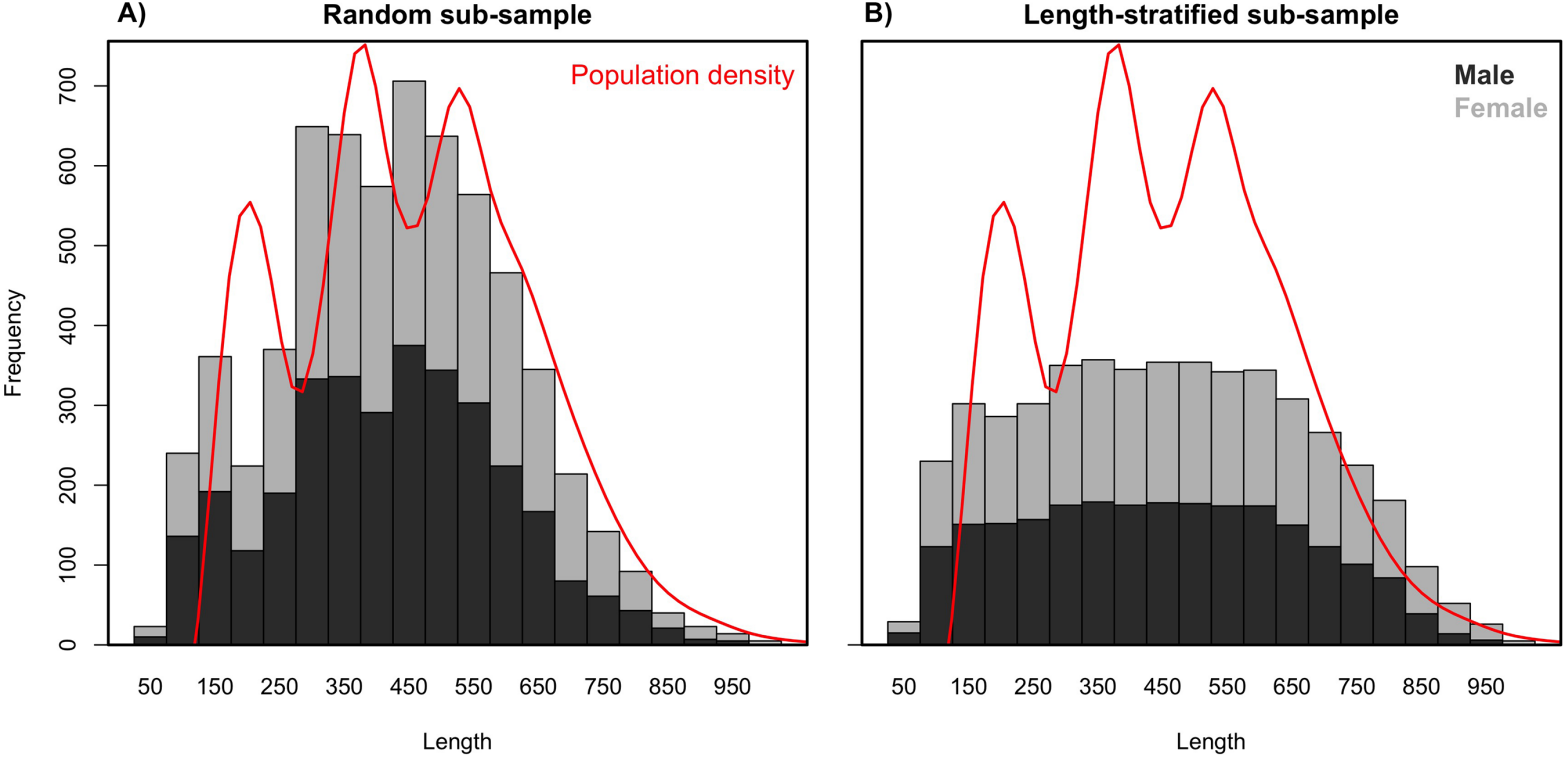
Length frequency distributions. Average length frequency distribution obtained with (A) the random and (B) the length-stratified sub-sampling designs, respectively, including all scenarios. Red line denotes population density.

Age data sub-samples obtained by the RS and LSS strategies differed in the number of records collected (stage 3) as a result of their respective intrinsic designs. While the random sub-sampling strategy accounted for ∼1,000 records per scenario including all the preselected sampling sites (stage 1), the LSS collected only ∼800, dismissing ∼5% (13–22) sampling sites per scenario as a result of the targeted number of otoliths having already been reached. As expected due to the differences in the designs, the random sub-sample showed a normal distribution for the length measurements (stage 2), where the most common lengths are more representative (Fig. 2). Naturally, the length-stratified sub-sample led to a uniform distribution where all length intervals are equally represented, with the exception of the extremes that are not commonly found in nature (Fig. 2).

**Figure 3:**
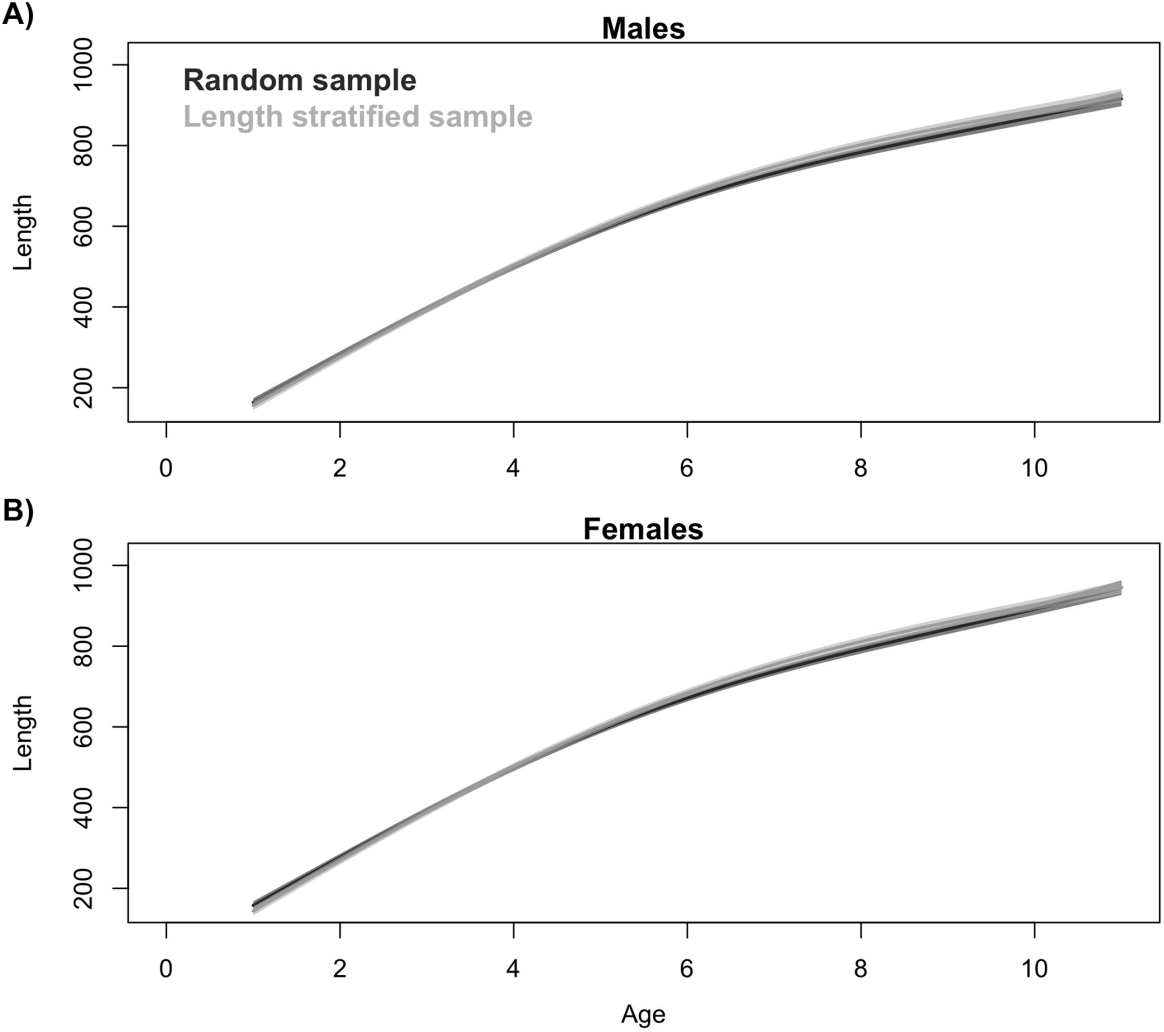
Trait distribution from sub-samples. Average age at size function extracted from the models parameterized in (A) the random and (B) the length-stratified sub-sampling strategy, respectively, including all scenarios. Only partial effect of size and sex are shown.

**Figure 4:**
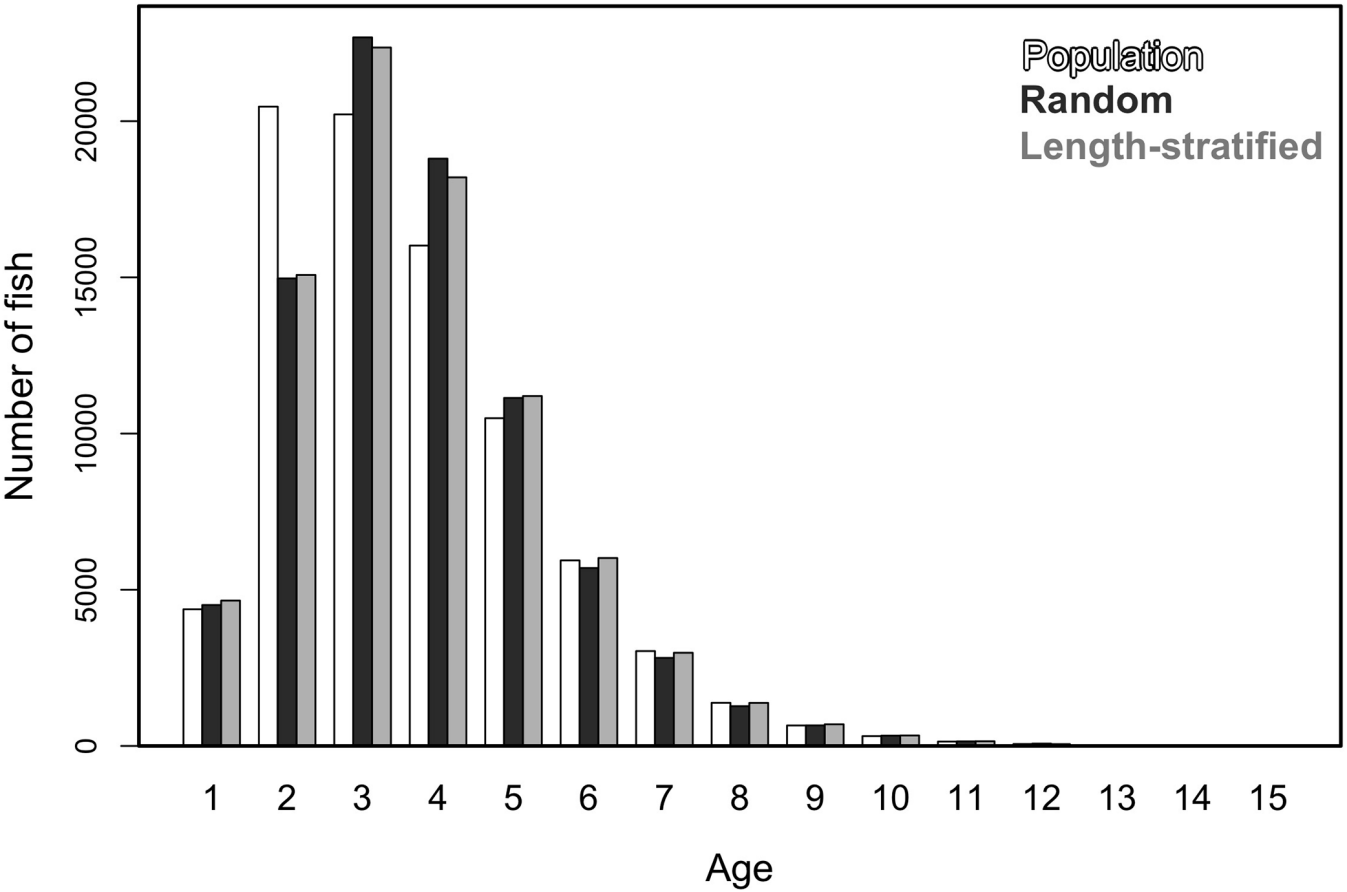
Trait frequency distribution from population and sub-samples. Age frequency distribution of the virtual population compared to that recovered from the random and length-stratified age sub-samples (including all scenarios).

**Figure 5:**
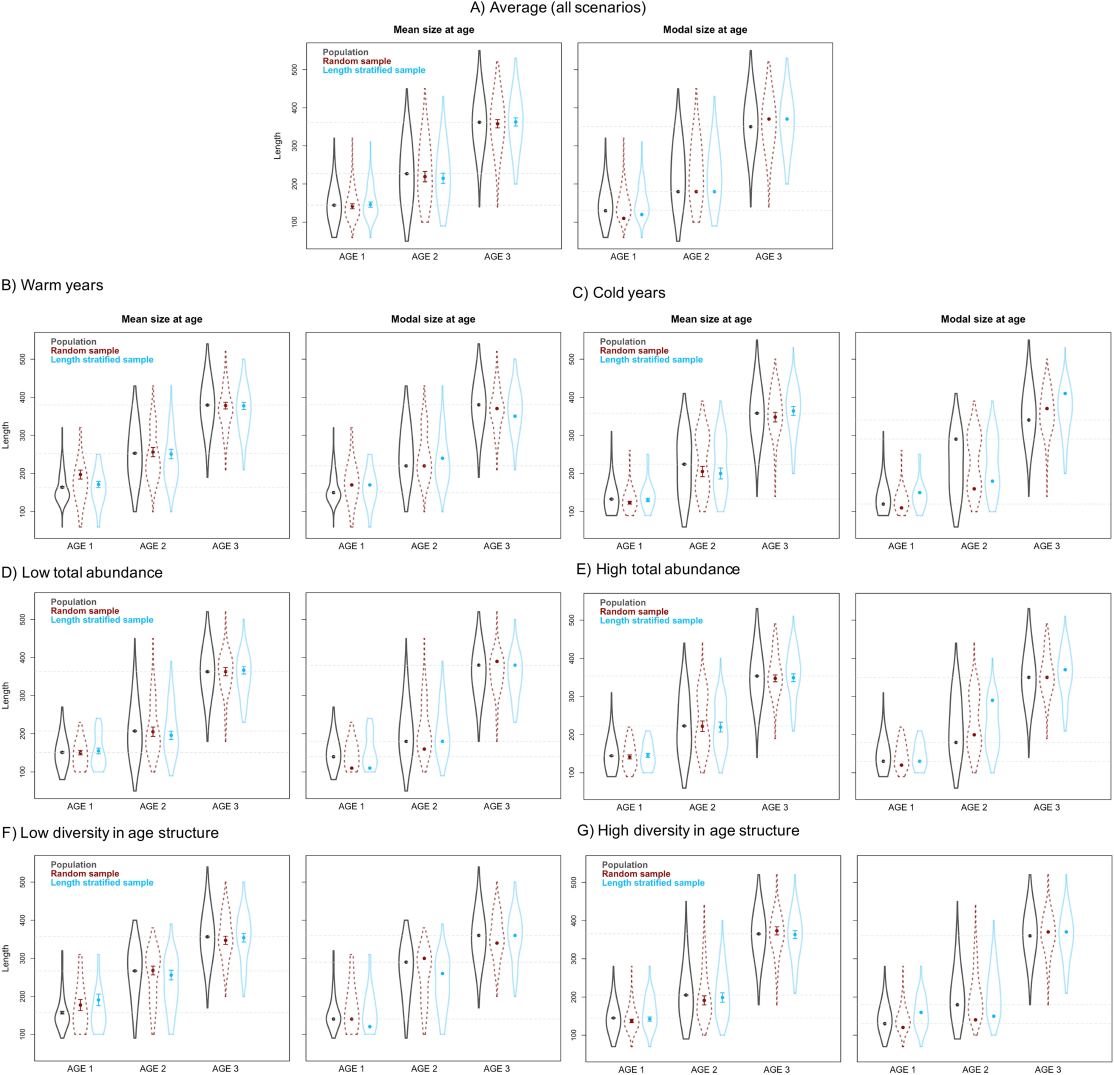
Mean and modal estimates of the population’s trait. Mean and modal size at age for age-groups 1–3 calculated for the virtual population (gray), the random (red) and length-stratified (blue) age sub-samples, respectively. (A) Average estimates combining all scenarios, (B) warm years, (C) cold years, (D) low total abundance, (E) high total abundance, (F) low diversity in age structure and (G) high diversity in age structure. Dots denote point estimates values, error bars show standard errors and violin contours the dispersion and density of the data used. Horizontal dashed line highlights virtual population values.

**Figure 6:**
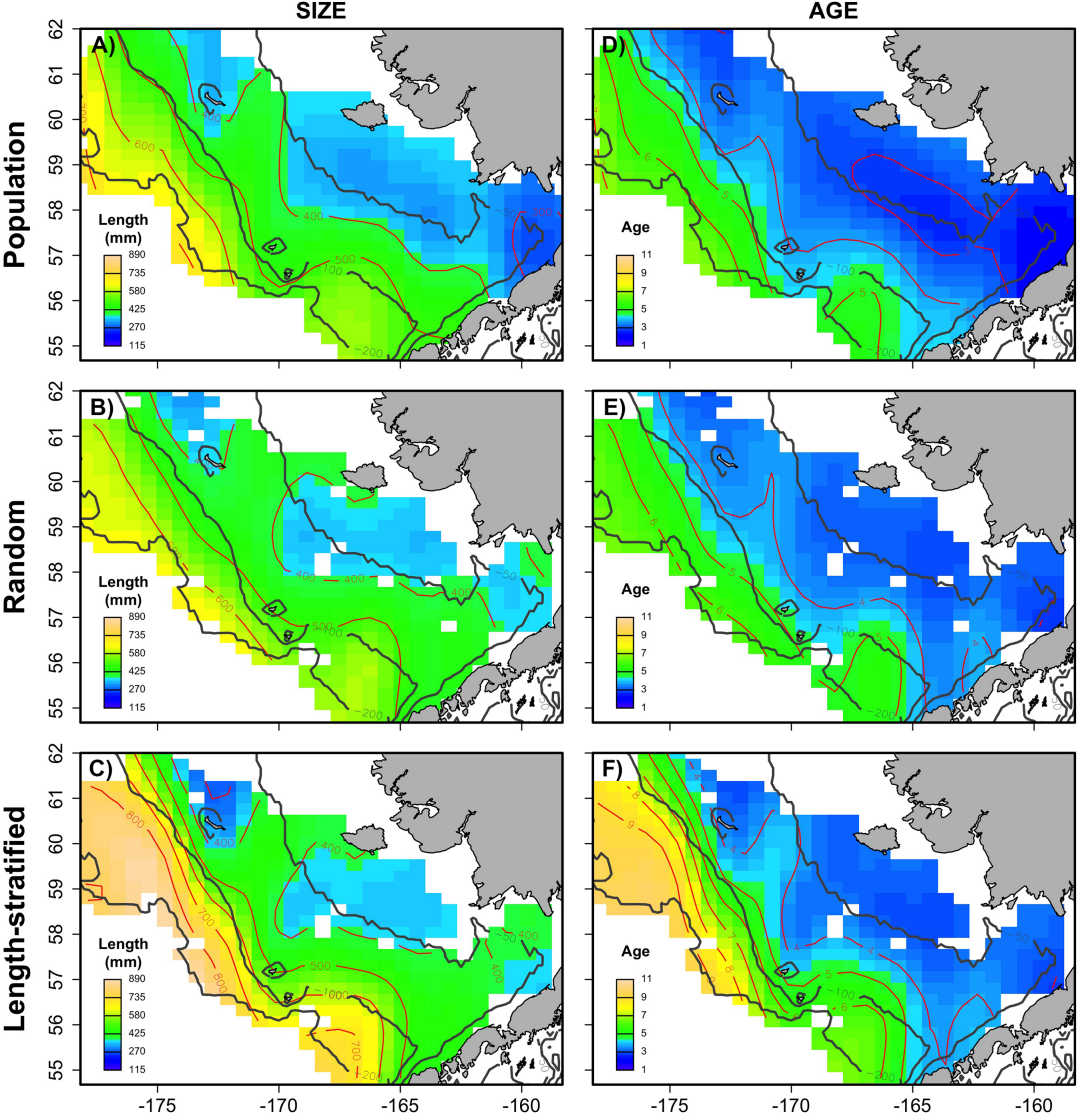
Spatial patterns of the trait obtained from sub-samples. Average spatial distribution of size (A–C) and age (D–F) for the virtual population, the random and length-stratified sub-sample, respectively. Gray contour lines define the bathymetry at 50, 100 and 200 m depths that divides the inner, middle and outer shelf. Red contour lines contours delimit spatial distribution for the corresponding values in the color scale.

- Generalized additive models parameterized on the RS and LSS age sub-samples showed minimal differences in terms of explained variability, prediction errors (Table 1) and functional shape in size at age (Fig. 3). These negligible differences were also observed at each individual scenario, but we observed greater standard deviation in predicted errors from the LSS strategy due to the lower sample size used. Model residuals were inspected for normality, homoscedasticity and independence. None of these model residuals exhibited strong spatial autocorrelation, indicating that the model formulation was sufficient to address the spatial autocorrelation incorporated in the virtual population (Fig. S3).
- Similar age frequency distributions were predicted from the two age sub-samples (Fig. 4). This was expected considering the negligible differences in size-at-age relationships. However, both sub-sampling strategies misrepresent the number of fish at the most representative age groups (ages 2, 3 and 4) and, particularly the age 2 group. This age accounted for ∼26% of the individuals misplaced in an age group regardless of the sub-sampling strategy used. Although shape and prediction of the age frequency distribution varied notably across the different individual scenarios (Fig. S2), the underestimation of the age 2 group was observed in all cases. The number of miss-aged individuals increased by 2% approximately in the individual scenarios comparing with the average.
- The average point estimates of mean and modal size at age derived from the RS and LSS age sub-samples were both almost identical to the values observed in the virtual population for age-groups 1–3 (Fig. 5A). However, a mismatch between population and sub-sample point estimates was frequent and sometimes remarkable in the individual scenarios, with the RS tending to provide more accurate and precise estimates (Fig. 5). The most diverging differences occurred in cold conditions and high diversity of age structure. Spatial distribution of cod in simulated scenarios also notably diverged between warm and cold years (Fig. S1).
- The largest and most remarkable differences between the RS and LSS sub-sampling strategies were observed in the recovery of the average spatial patterns in size and age (Fig. 6). The length-stratified sample was not able to capture the spatial patterns in the virtual population and largely overestimated these patterns in shape and particularly, in magnitude. Larger and older fish estimated by the LSS were disproportionally widely distributed in the northwest region of the outer shelf, presenting an average age of 3 years older than the age observed in the virtual population. By contrast, the RS strategy recovered very similar patterns as those in the virtual population. Despite the fact that the population distributional patterns considerably varied between the different individual scenarios, the misrepresentation described for the LSS was observed in all cases (Fig. S4).

**Table 1:** Comparisons of models with and without spatial term.

Model	% Variance	Error	sd
Random	89	0.65	0.11
Stratified	92.2	0.66	0.11
Random (no spatial)	87.5	0.68	0.12
Stratified (no spatial)	91.3	0.69	0.11

**Note:**

Model comparisons including percentage of explained variance, mean prediction error and its standard deviation. Random and Stratified models were formulated as in Eq. (2), while the no spatial models used this formulation but removing the spatial term.

### Sensitivity test

**Figure 7:**
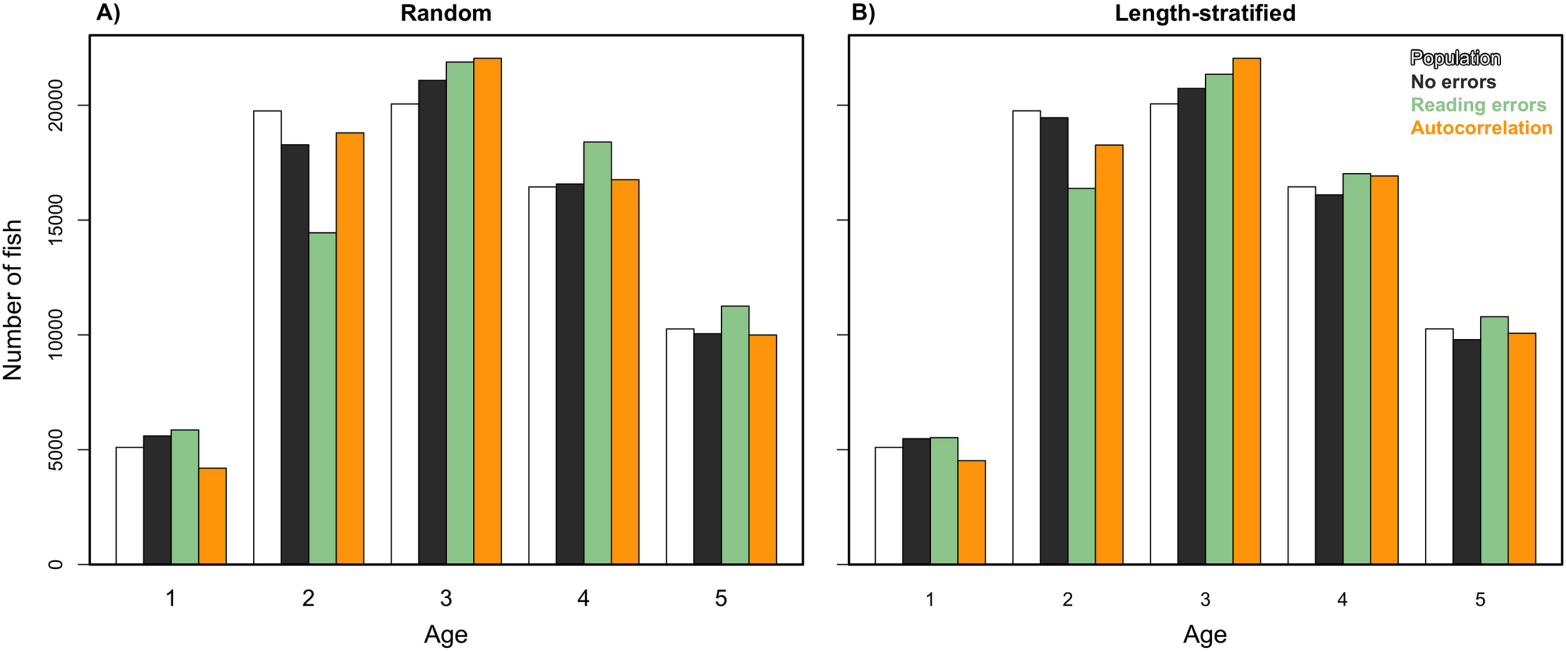
Sensitivity test: predicted values of the trait. Age frequency distribution predicted from (A) the random and (B) the length-stratified sub-samples, respectively, including all the scenarios, and every combination of the different errors included along the simulation framework.

We analyzed models parameterized in the RS and LSS, respectively, as in Eq. (2) but removing the spatial term. Results indicated that spatial location had a small contribution in the variance explained and modestly reduced the predictability skills (Table 1). However, this small reduction turned into large differences when comparing the predicted age distribution (number of fish per age group) with and without including the spatial term in the model (Eq. (2)). The effects of incorporating different errors, such as autocorrelation and reading bias and error were mainly detected in the predicted age distribution. Autocorrelation errors in the virtual population did not affect the predictive ability of the models, since as mentioned, it was properly addressed by the spatial term in the model formulation. However, the autocorrelation error contributed to ∼13% of the fish being mis-aged (Fig. 7). Removing the reading bias and error resulted in the mis-aged number of fish to approximately be 5% (with a larger reduction in RS than in LSS, Fig. 7).

**Figure 8:**
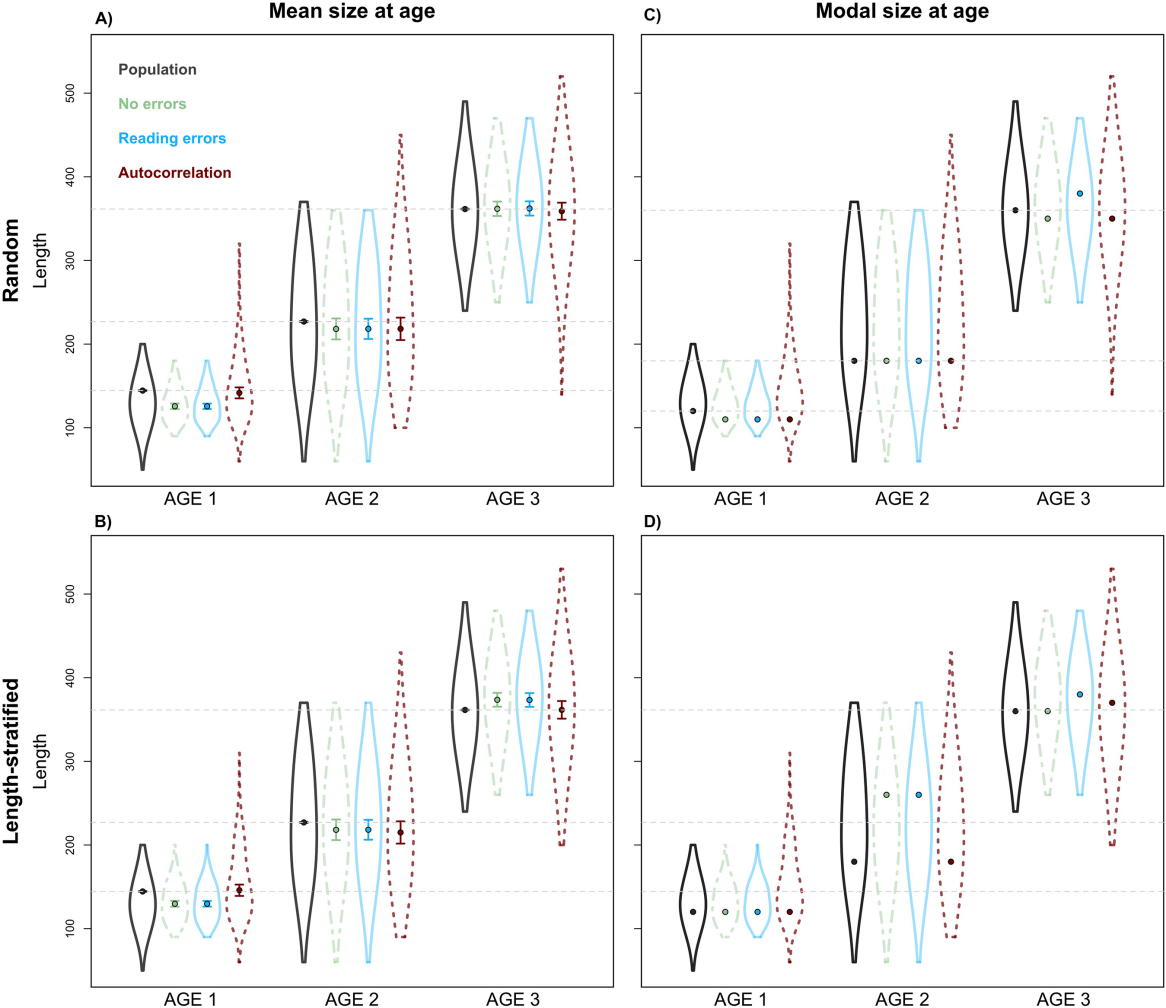
Sensitivity test: mean and modal estimates. Mean (A–B) and modal (C–D) size at age for age groups 1–3 calculated for the virtual population (gray), the random and length-stratified age sub-samples under different sensitivity test: none error (green), effect of effect of bias and error of age data processing (blue) and spatial autocorrelation (red). Dots denote point estimates values, error bars show standard errors and violin contours the dispersion and density of the data used. Horizontal dashed line highlights virtual population values.

Unexpectedly, this variation in the predicted number of fish at a given age did not have a notable effect on the point estimates for mean and modal size at age (Fig. 8). Nevertheless, a mismatch in the point estimates between population and age sub-sample values can be an issue associated with small sample size in the RS and LSS sub-samples. Indeed, when we increased the sub-sample size to all fish caught (stage 3, i.e., not random or length-stratified sub-sampling either), the points estimates match perfectly between the population and the age sub-sample data (results not shown). The sampling strategy evaluation provide the same results when considering LSS and RS subsamples with the same total sample size (results not shown).

## Discussion

This study provides a useful framework to simulate varying populations with spatially structured traits and assess the precision and performance of different sub-sampling strategies for monitoring biological populations in a multistage design. The sub-sampling strategy evaluation showed that the population parameters estimated with the random and the stratified sub-sample, (e.g., prediction error, mean and modal trait values) were almost negligible between the two sub-sampling strategies when multiple scenarios (i.e., increasing sample size and variability in the population) were considered as input data. However, noticeable differences were found between the average spatial distribution of individual traits of the virtual population and those reconstructed from the LSS sub-sampling (Fig. 6). This design resulted in a spatially uneven collection of the sub-samples that prevent the recovery of the geographical patterns of the population. Similar results were observed in the field (Puerta et al., 2018), where differences between the sub-sampling strategies were reduced when considering survey data from multiple years.

In the particular case of the EBS Pacific cod monitoring program, the LSS is used in the surveys, which start in the SE inner-middle shelf, then move toward the NW region of the EBS until the target sub-sample size (i.e., three fish per sex and length) is reached (Conner & Lauth, 2017). Thus, the LSS sub-sample (stage 3) is collected predominantly the SE area. In contrast, the NW area is less represented in the sub-sample size, despite the attempt to evenly spread sampling sites (stage 1) between the two regions. Considering three sampling sites located from SE to NW and pre-selected for age data sub-sampling, the simulation showed that the RS collected four individuals in each site, while LSS collected 28, one and zero in these same sites. In addition, meaningful improvements were detected in favor of the random design when analyzing individual scenarios, that is, corresponding to an isolated survey of a particular population. In this situation, the random sub-sampling strategy provided not only an accurate representation of the average spatial patterns of the studied trait, but also achieved higher precision in the population estimates. These conditions were particularly acute during scenarios characterized by cold years and low age diversity. We demonstrated that the accuracy in the estimates derived from the RS are not related with the larger sample size obtained with this design, but with its inherent characteristics, such as the even spatial coverage. Achieving precise population estimates across all conditions is particularly important for the management of natural populations, as occurred for instance in fisheries, where the assessment and thus, management decisions, are conducted on a yearly basis. The random design is a more practical and time-saving option to implement in the field studies and provides wider and more even coverage of data to be used as input for geostatistical mapping and analysis. That might be particularly relevant advantage for marine studies, where pre-survey information is very challenging to achieve. In terrestrial research, technology tools such as remote sensing provide pre-survey information on habitats, population patchiness or other characteristics that permit reliable stratification of the study area and the population to improve survey precision (McGarvey, Burch & Matthews, 2016), while only surface layer information can be obtained in marine systems, thus challenging an efficient and biologically meaningful stratification.

Sensitivity analysis allowed us to disentangle error sources and their contributions that should not be confounded with the intrinsic differences in the design of the sub-sampling strategies. As expected, part of the predictive error arose from the technical procedure used to extract information on the sub-sample trait (i.e., the otolith reading process in the study case). However, a much larger contribution to the error estimates derived from the spatial autocorrelation in the virtual population. Spatial autocorrelation is rarely considered in the analysis of individual traits in natural population studies (Goslee, 2006; McGarvey, Burch & Matthews, 2016). However, we demonstrated the important contribution of spatial autocorrelation in the misclassification of individual ages due to an amplification of the error in a particular group. In our study case, the abundance of Pacific cod age 2 group was underestimated in all scenarios (except in the simulation with no errors), despite the fact that the reading bias and reading error are among the lowest for this age-group. The quadratic size at age function (Eq. (1)) used to create the virtual population trait structure, makes the age 2 group the most sensitive to errors. Therefore, when autocorrelation errors are added, the effect is widely amplified in this age group resulting in large underestimation of this age group by both of the sub-sampling designs.

## Conclusions

We have developed a modeling framework that will be a useful tool for monitoring and assessment of natural populations since the simulations and the sampling strategy evaluation can potentially address multiple methodological issues in a simple, fast and low-cost way under a completely controlled environment. Sampling designs are of paramount importance in scientific surveys and an inappropriate selection of the sampling and sub-sampling methods might lead to biased and inaccurate results that do not support reliable management advice (Anderson, 2001; Legg & Nagy, 2006). Beyond our study case in size at age estimates in fisheries, there are potentially more applications in other fields, environments and ecological estimates that depend on spatial patterns of populations such as maturity in terrestrial animals, CO_2_ fluxes in forest, prey consumed by predators.

## Supplemental Information

10.7717/peerj.6471/supp-1 Supplemental Information 1 Pacific cod 1994–2016 catch data. Each data point represent a haul in historical series 1994–2016 Eastern Bering Sea bottom trawl survey.Data includes date, time, locations, depth, bottom temperature and catch in number of fish and weight. Every haul has a unique code included as ‘hauljoin’.

10.7717/peerj.6471/supp-2 Supplemental Information 2 Pacific cod length samples. Each data point represent the frequency of fish caught at a particular length and sex for every haul in the catch data.

10.7717/peerj.6471/supp-3 Supplemental Information 3 Pacific cod otolith sub-samples. Each data point represent an individual fish from the Eastern Bering Sea bottom trawl survey (1994–2016) that was caught, sexed, lengthened and aged.

10.7717/peerj.6471/supp-4 Supplemental Information 4 Spatial distribution of abundance, size and age of the virtual population, showing average patterns in every individual scenario. a) Warm years, b) Cold years, c) Low total abundance, d) High total abundance, e) Low diversity in age structure and f) High diversity in age structure.

10.7717/peerj.6471/supp-5 Supplemental Information 5 Age frequency distribution of the virtual population compared to that recovered from the random and length-stratified age sub-samples in every individual scenario. a) Warm years, b) Cold years, c) Low total abundance, d) High total abundance, e) Low diversity in age structure and f) High diversity in age structure.

10.7717/peerj.6471/supp-6 Supplemental Information 6 Residuals and model checking of GAMs in Figure 3. A) GAM fitted on random subsample data. B) GAM fitted on random subsample data.

10.7717/peerj.6471/supp-7 Supplemental Information 7 Average spatial distribution of size and age for the virtual population, the random and length-stratified sub-sample, respectively, in every individual scenario. a) Warm years.

10.7717/peerj.6471/supp-8 Supplemental Information 8 Average spatial distribution of size and age for the virtual population, the random and length-stratified sub-sample, respectively, in every individual scenario. b) Cold years.

10.7717/peerj.6471/supp-9 Supplemental Information 9 Average spatial distribution of size and age for the virtual population, the random and length-stratified sub-sample, respectively, in every individual scenario. c) Low total abundance.

10.7717/peerj.6471/supp-10 Supplemental Information 10 Average spatial distribution of size and age for the virtual population, the random and length-stratified sub-sample, respectively, in every individual scenario. d) High total abundance.

10.7717/peerj.6471/supp-11 Supplemental Information 11 Average spatial distribution of size and age for the virtual population, the random and length-stratified sub-sample, respectively, in every individual scenario. f) Low diversity in age structure.

10.7717/peerj.6471/supp-12 Supplemental Information 12 Average spatial distribution of size and age for the virtual population, the random and length-stratified sub-sample, respectively, in every individual scenario. f) High diversity in age structure.

## Additional Information and Declarations

### Data Availability

The following information was supplied regarding data availability: Raw data are available in the Supplemental Files.
