# Supplementary figures and images for: A simulation framework for evaluating multi-stage sampling designs in populations with spatially structured traits
Source: PeerJ. 2019 Feb 25;7:e6471. doi: 10.7717/peerj.6471 (PMC6394348; doi:10.7717/peerj.6471)

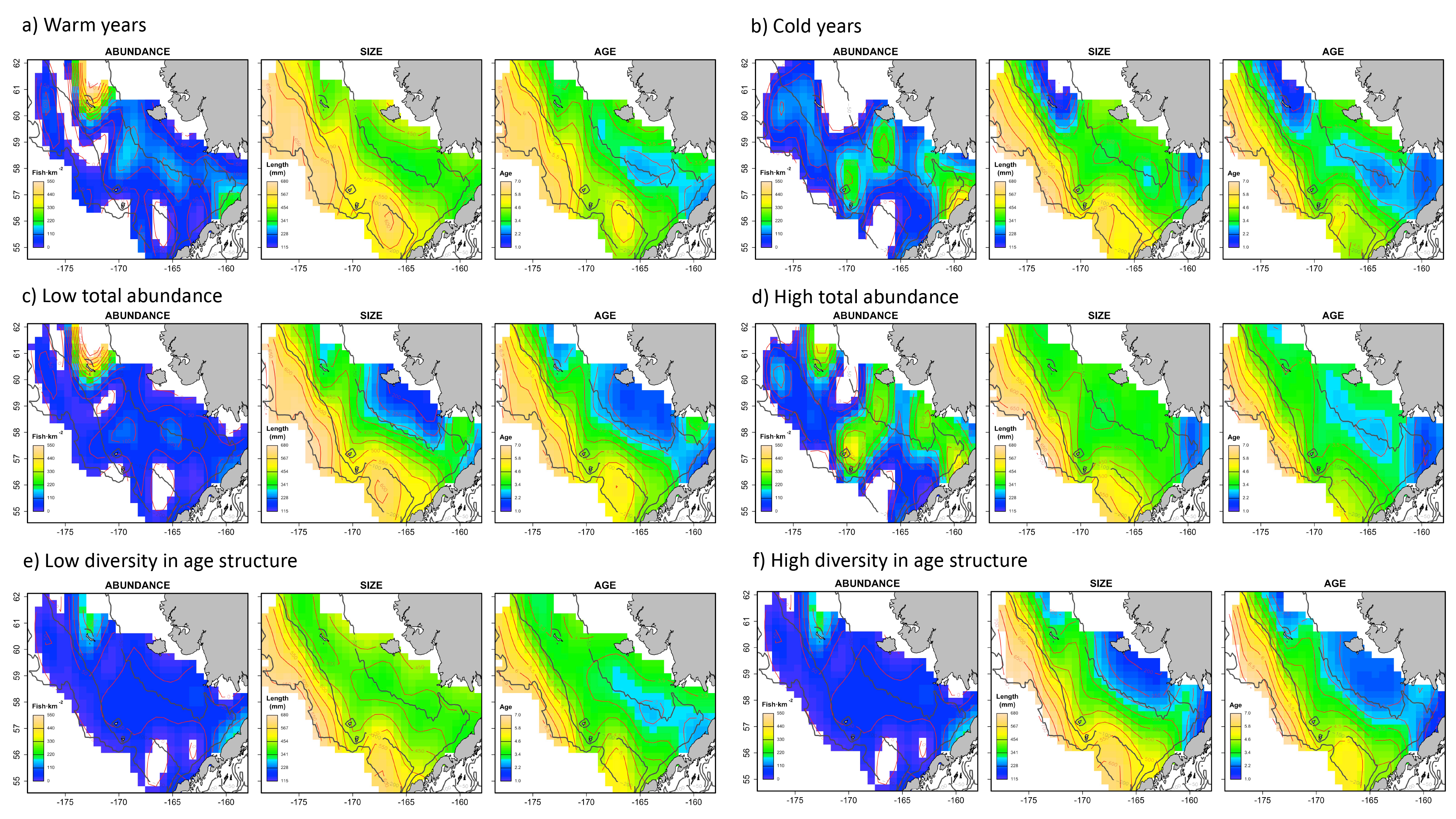

Supplement: Supplemental Information 4 — a) Warm years, b) Cold years, c) Low total abundance, d) High total abundance, e) Low diversity in age structure and f) High diversity in age structure. [file peerj-07-6471-s004.png]

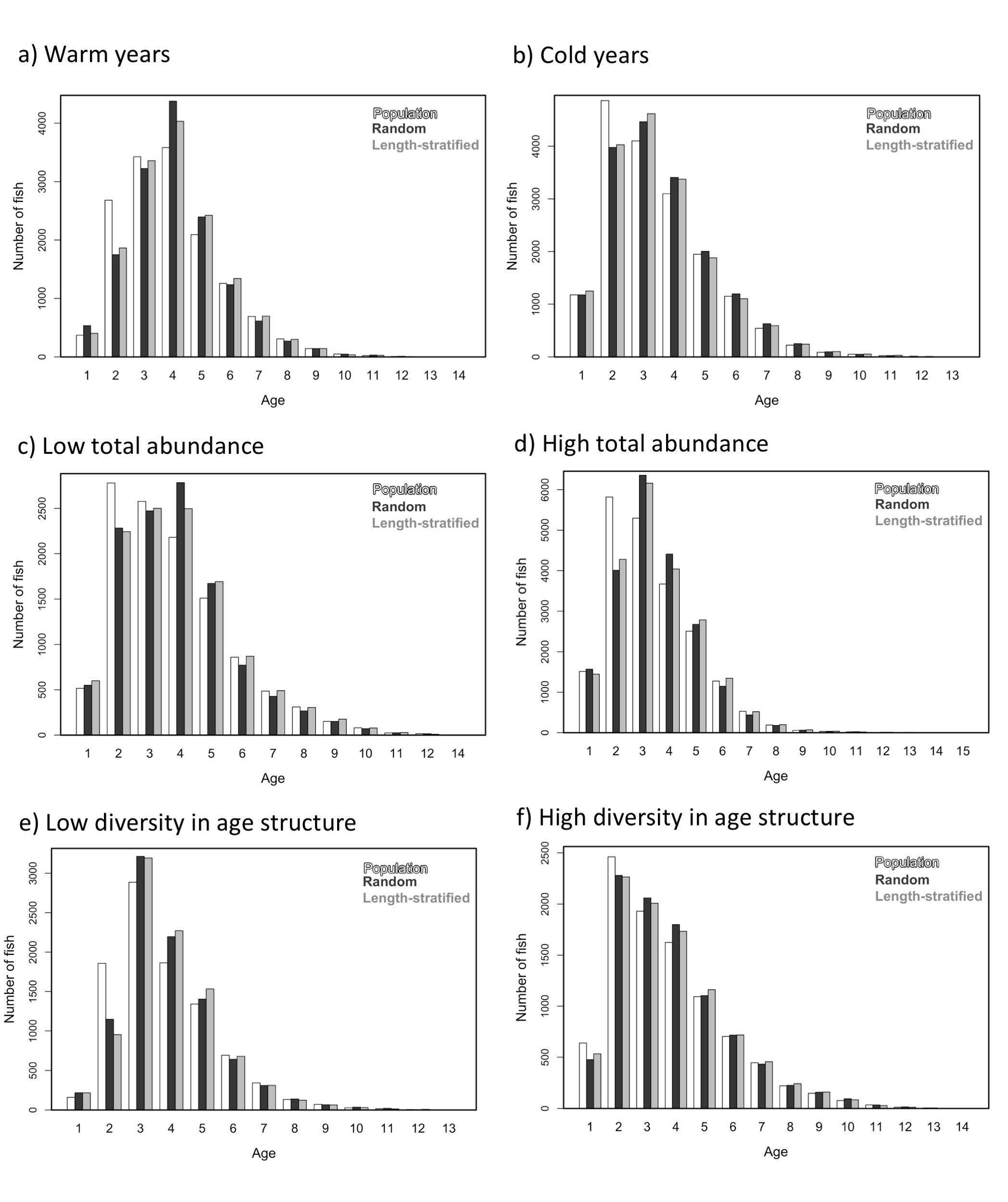

Supplement: Supplemental Information 5 — a) Warm years, b) Cold years, c) Low total abundance, d) High total abundance, e) Low diversity in age structure and f) High diversity in age structure. [file peerj-07-6471-s005.png]

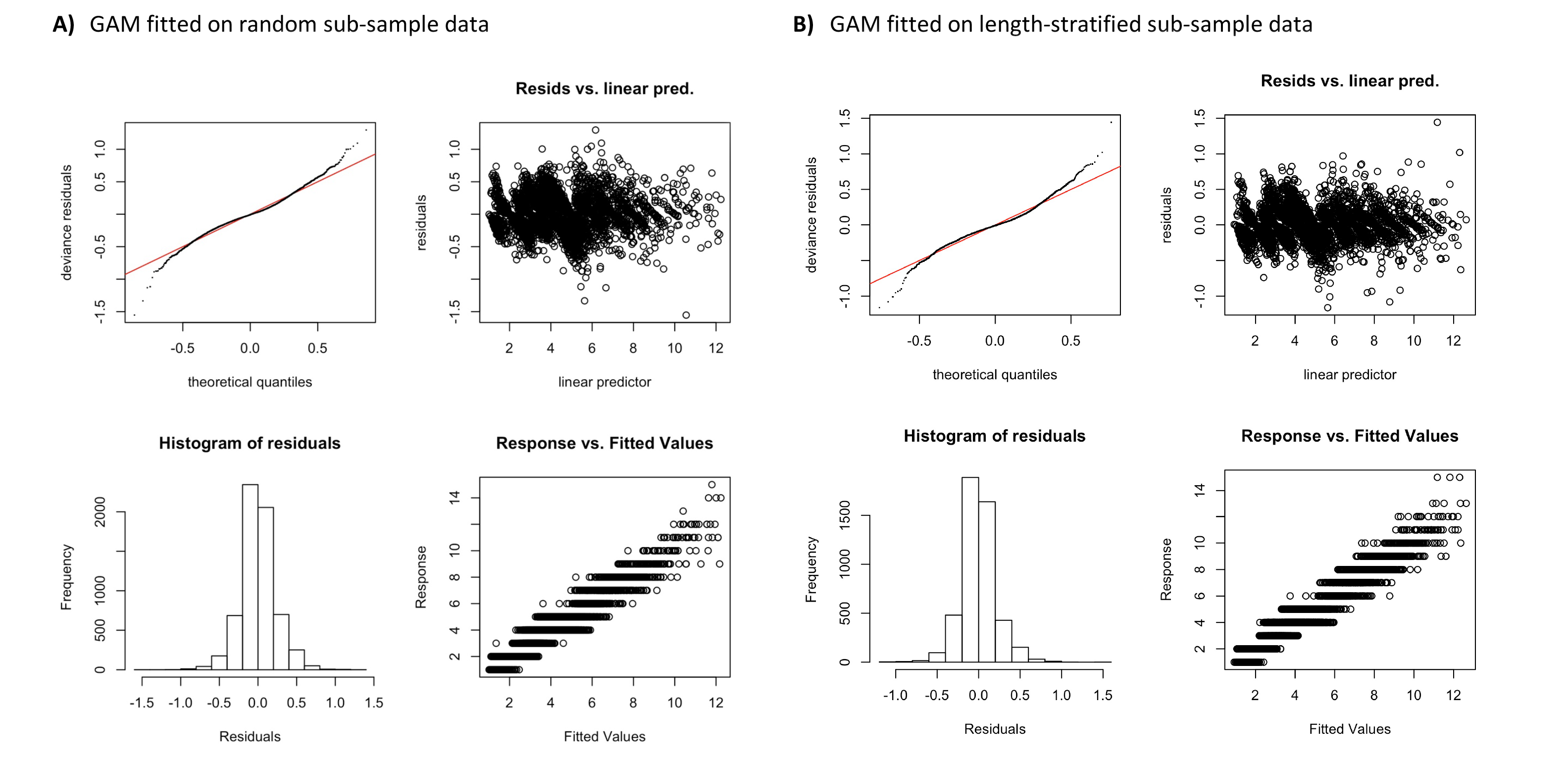

Supplement: Supplemental Information 6 — A) GAM fitted on random subsample data. B) GAM fitted on random subsample data. [file peerj-07-6471-s006.png]

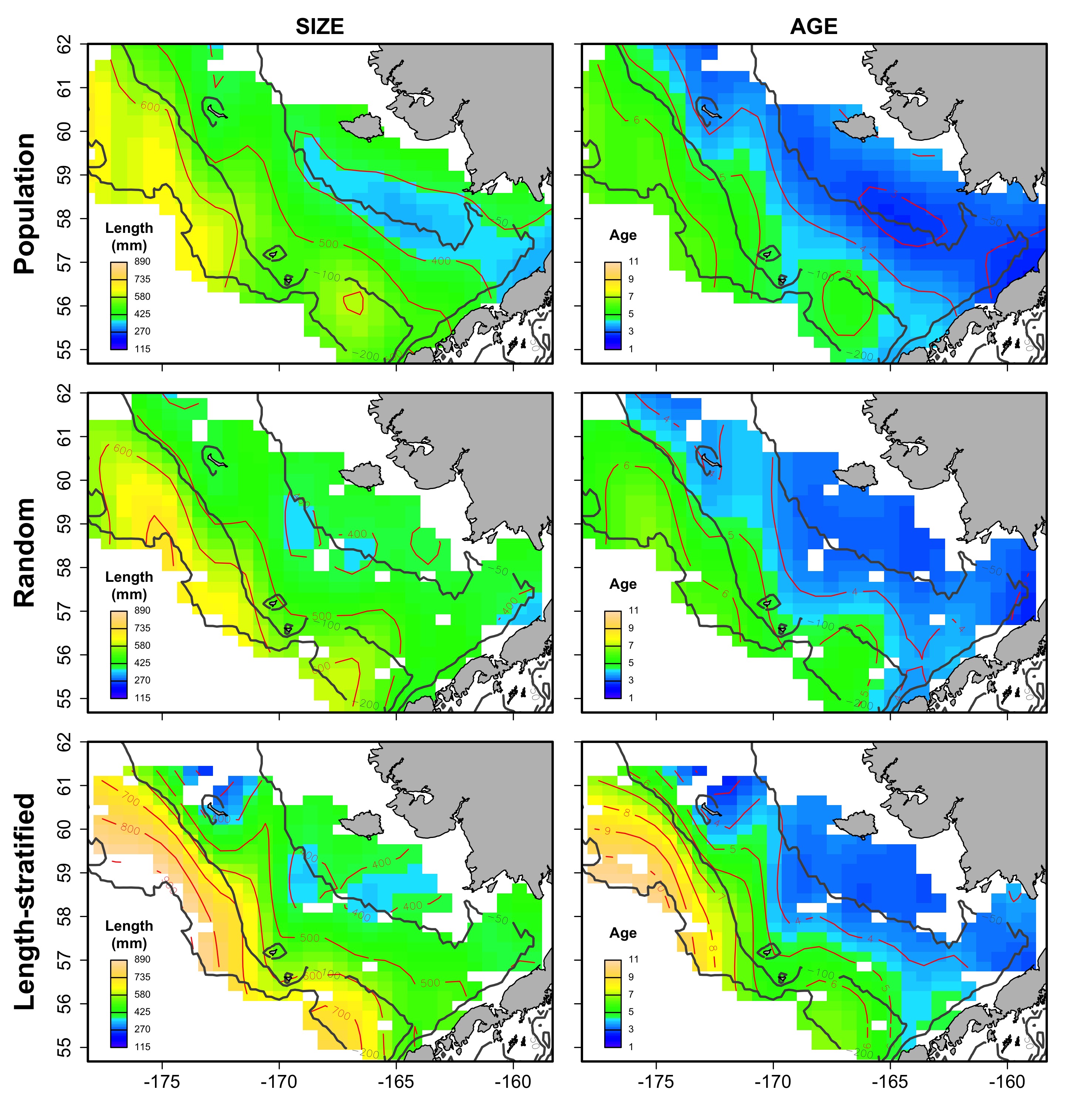

Supplement: Supplemental Information 7 — a) Warm years. [file peerj-07-6471-s007.jpg]

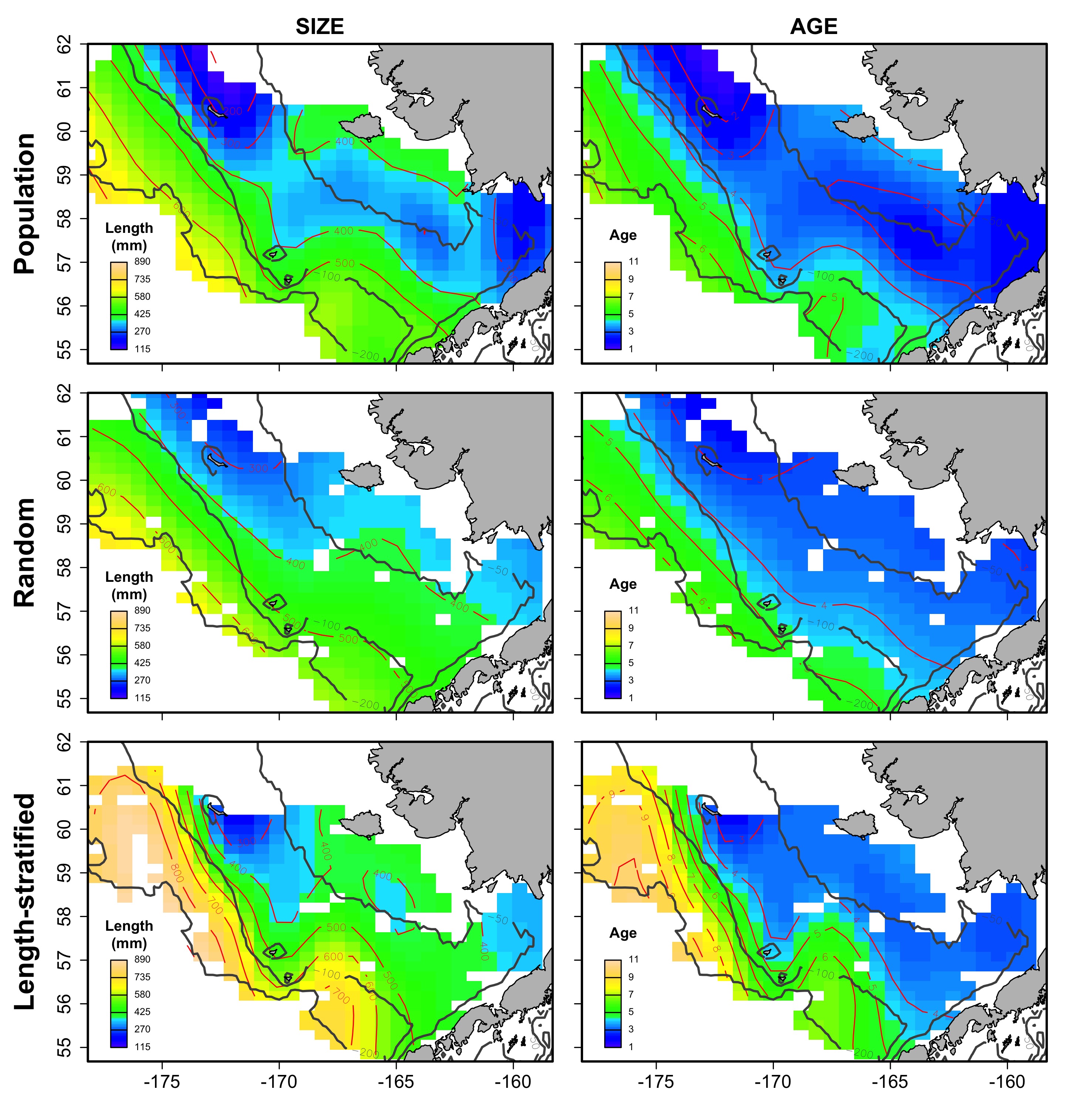

Supplement: Supplemental Information 8 — b) Cold years. [file peerj-07-6471-s008.jpg]

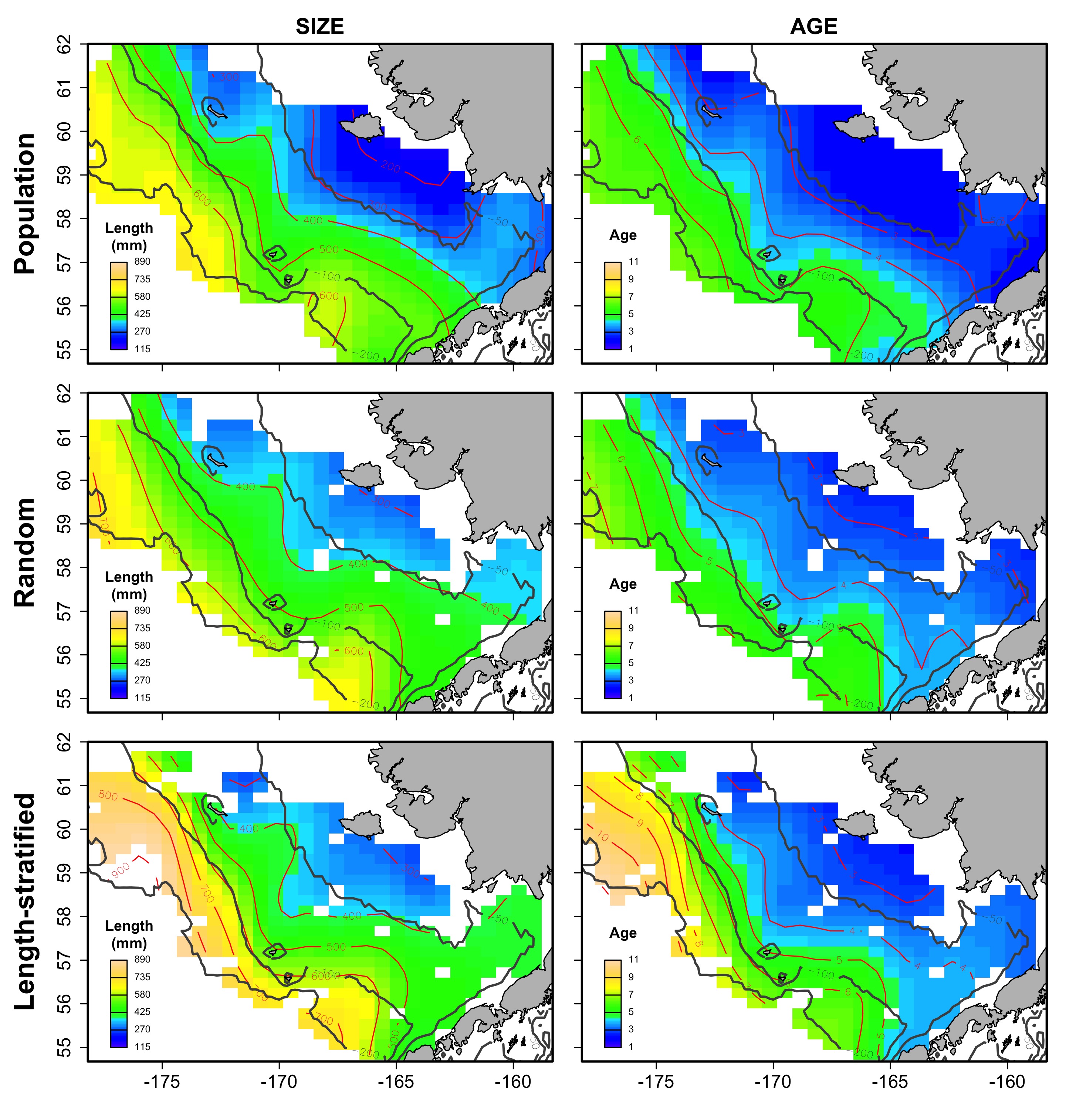

Supplement: Supplemental Information 9 — c) Low total abundance. [file peerj-07-6471-s009.jpg]

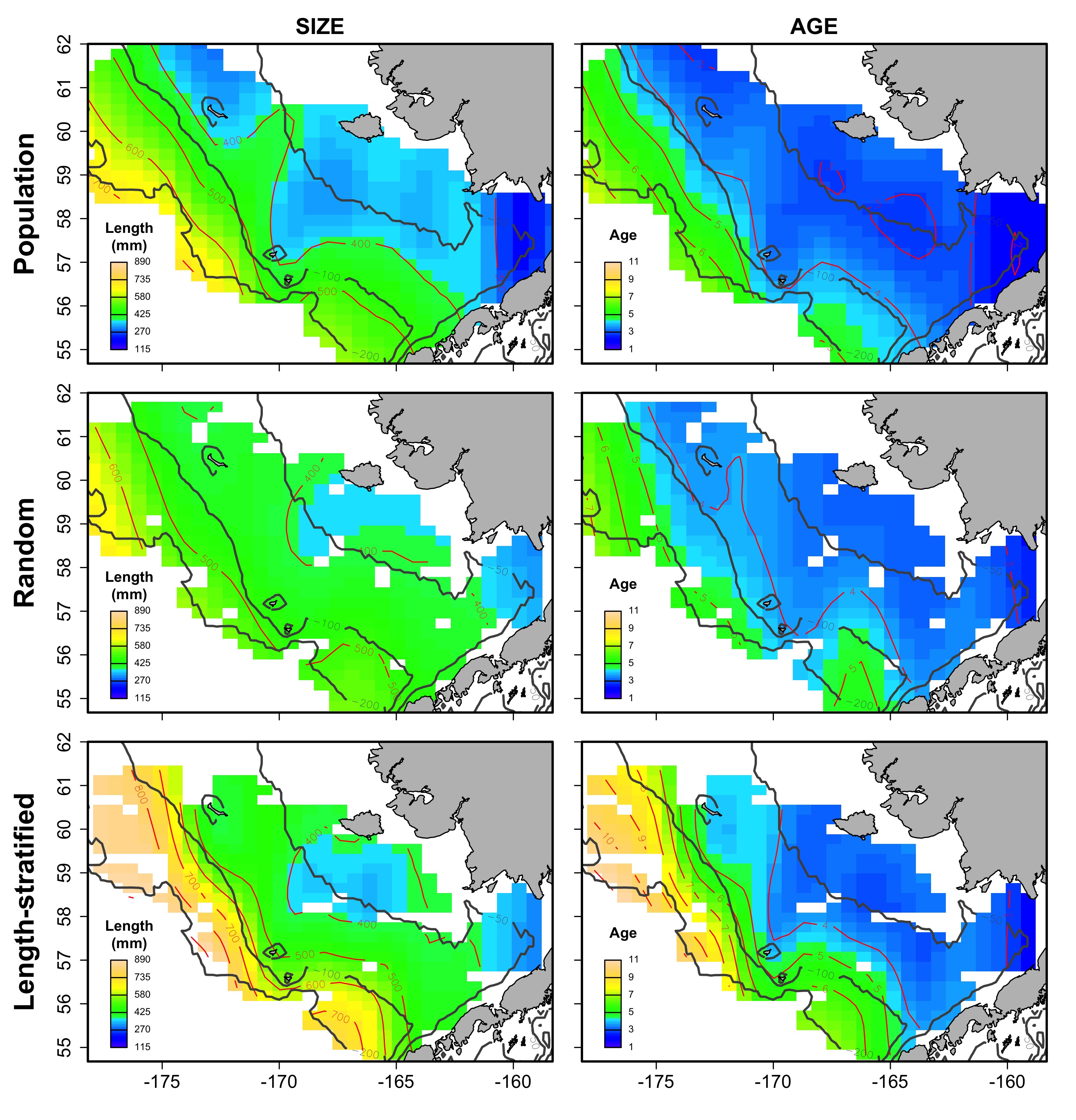

Supplement: Supplemental Information 10 — d) High total abundance. [file peerj-07-6471-s010.jpg]

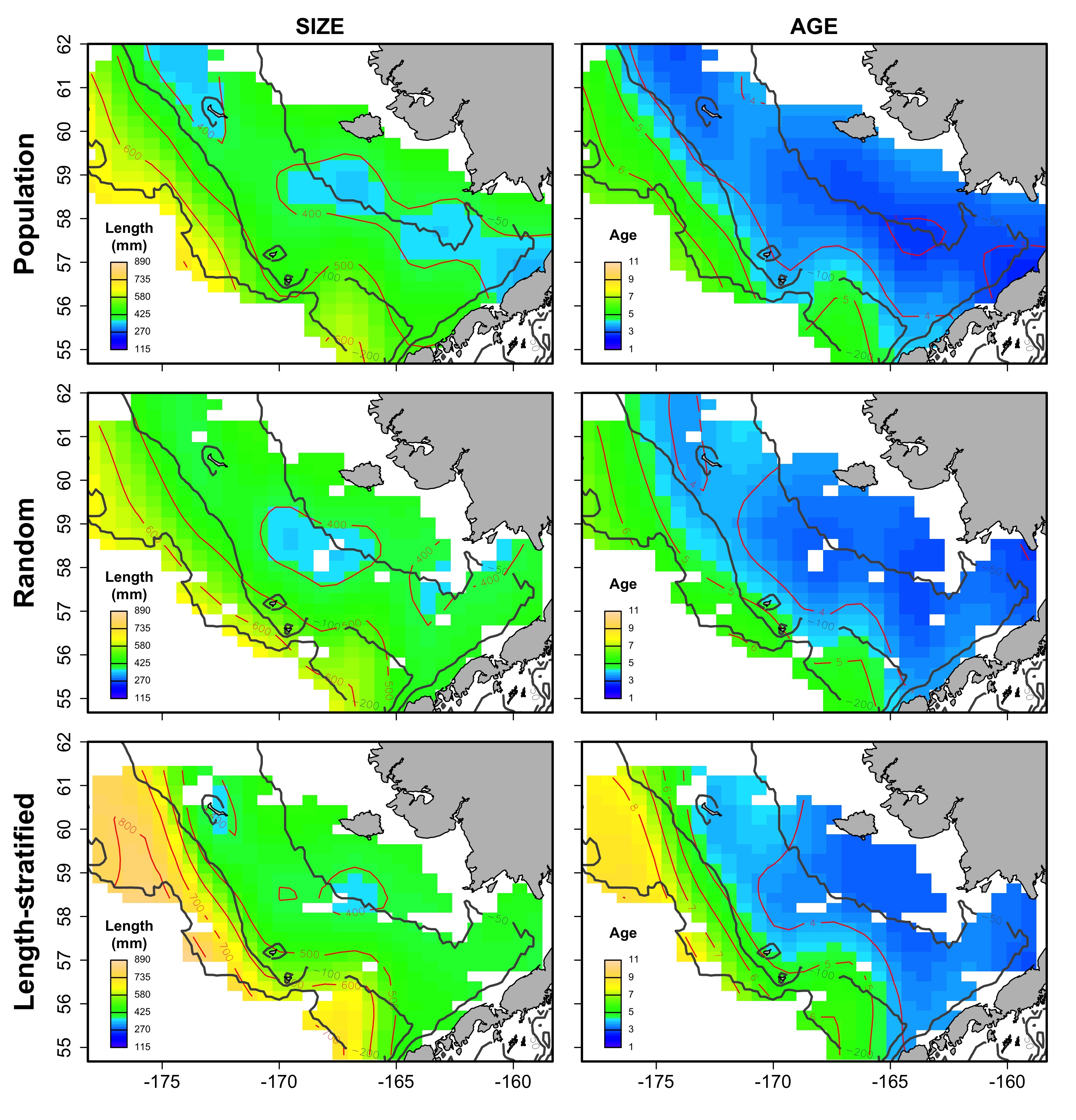

Supplement: Supplemental Information 11 — f) Low diversity in age structure. [file peerj-07-6471-s011.jpg]

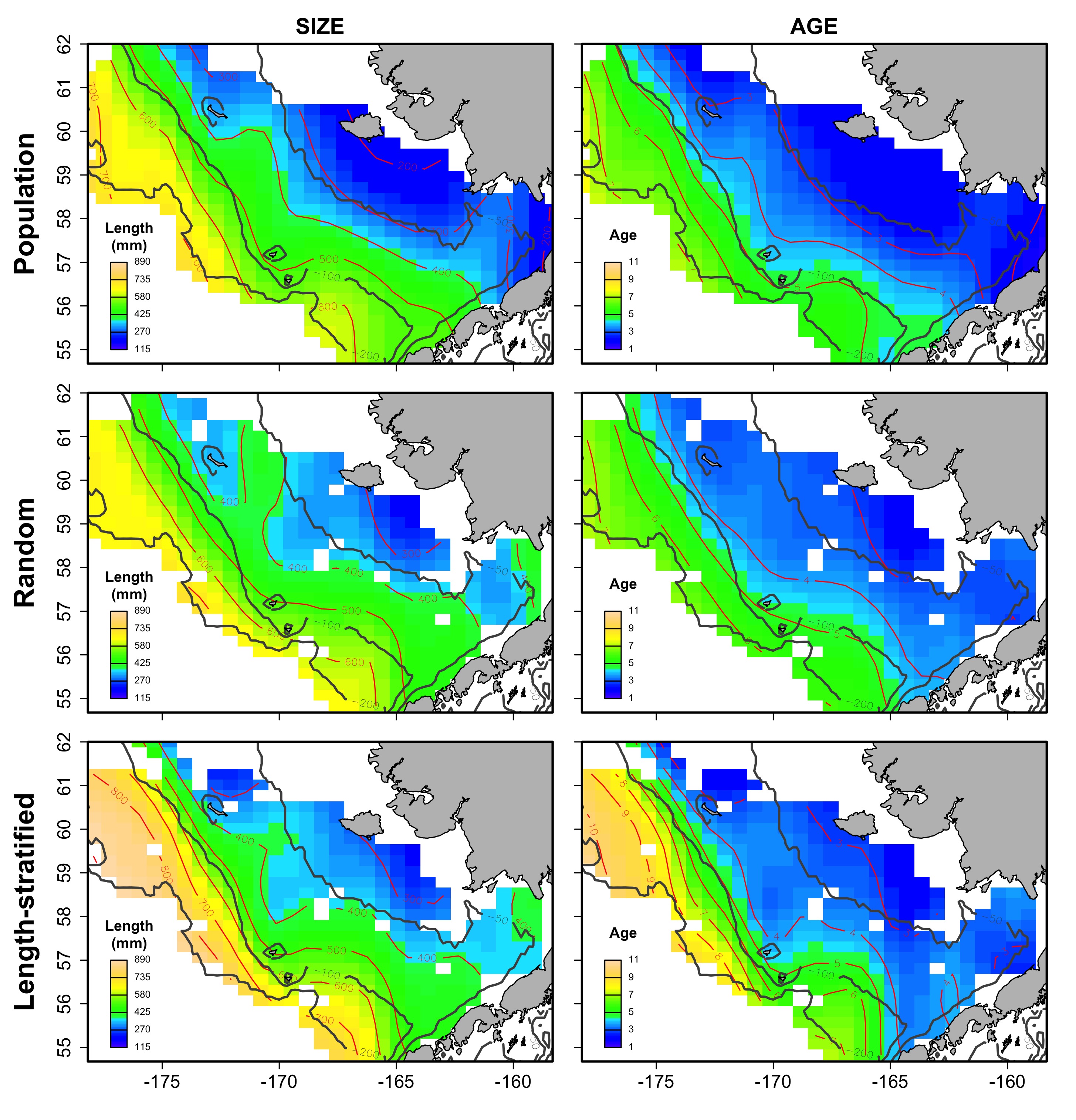

Supplement: Supplemental Information 12 — f) High diversity in age structure. [file peerj-07-6471-s012.jpg]
